# The attitude towards disclosure of bad news to cancer patients in Saudi Arabia

**DOI:** 10.4103/0256-4947.60520

**Published:** 2010

**Authors:** Ali H. Aljubran

**Affiliations:** From the Section of Medical Oncology, Oncology Center, King Faisal Specialist Hospital and Research Center, Riyadh, Saudi Arabia

## Abstract

Disclosing the diagnosis or prognosis to cancer patients in Saudi Arabia can be a serious challenge to the physician in his daily clinic practice. The public attitude towards full disclosure is still conservative, and in order to appropriately deal with such an attitude, physicians need to deeply understand its sociocultural background. This article attempts to look into what governs the public attitude towards disclosure in Saudi Arabia as an example of what may affect attitudes in developing countries. It also brings some data from local surveys among physicians and patients as well as from public surveys to describe the changing trend in attitude over the years with a comparative analysis of the Western literature.

During the daily clinic practice, physicians occasionally find themselves confronted with situations of clear contradiction between what they have learned as the respectable ethics of medicine and what the public attitude dictates. In most of these situations, the family's request for non-disclosure of diagnosis and prognosis to the patient is the most contradicting and at the same time, the most difficult to manage. To appropriately respond to such a request, physicians need to deeply understand the sociocultural background of such an attitude.

This article attempts to look into what governs the public attitude regarding disclosure of full information to the patient in Saudi Arabia, as an example of the issue in many developing countries, and whether this attitude is amenable to the various psychosocial, educational influences or not.

Attitude is a hypothetical construct that represents an individual's degree of like or dislike for an item. Attitudes are generally positive, negative or neutral views of an “object”: i.e. a person, behavior or event.^1^ Most attitudes in individuals are a result of observational learning from their environment. The important question of whether attitudes change or not, was positively answered by many psychologists and socialists. Unlike personality, attitudes are expected to change as a function of experience. They can be changed through the tools of persuasion or by social influence like social proof and authority.^1^

## The attitude in western societies in the past

The general attitude among physicians in the West in the recent past was not in favor of fully discussing the diagnosis or prognosis with patients. In 1953, a questionnaire administered to 442 Philadelphia physicians regarding the issue of disclosure of diagnosis found that only 31% of the physicians surveyed stated that they always tell their patients the details of diagnosis.^2^ In 1960, a survey of 5000 physicians found that only 16% stated that they always tell the patients.^3^ Another survey one year later of 219 physicians in Chicago found that 90% stated that they generally do not inform patients of their diagnosis.^4^

On the other hand, the public attitude was not so different. In 1948, a public survey for the American Cancer Society covered many aspects of public reaction to cancer. Information was collected by personal interviews of 1244 adult persons. To monitor for changes in public opinions, two repeat surveys were conducted in 1955 and 1962. One relevant question was “Supposing that a doctor finds out that a person has cancer, should the person be told?” Percentages of response with a clear yes were only 63%, 64% and 60% in the years of 1948, 1955 and 1962, respectively.^5^

There was a combined medical and largely sociocultural background for such a conservative attitude towards disclosure. This background constitutes many elements including:

The nature of the disease. Cancer was, and still is in many situations, viewed as a death sentence, and revealing the diagnosis to a patient was considered cruel and inhumane. Patients' relatives thought that disclosure would lead to loss of hope and that the patient would become devastated and crippled or even die earlier, if told about the diagnosis.

Paternalistic medicine and the principle of beneficence helped physicians to collude with the patient's relatives in not explaining the status and prognosis of the disease to the patient in many settings.

The extended family role in the face of a less patient autonomy, in the past and to some extent in the present time, in many non-Western countries, is a major component of the sociocultural background of such an attitude. Patients were viewed as extended family, and all decisions, including health-related decisions, are family-centered decisions.

## The changing attitude: From 1960s and onwards

It is obvious that the attitudes of both physicians and the public have undergone major changes in the West in the past 50 years. In a questionnaire that was administered to a group of physicians who attended the 1999 Annual Meeting of the American Society of Clinical Oncology, participants were asked about difficulties they had when approaching stressful discussions and communication strategies used in giving unfavorable information.^6^ The questionnaire was completed by 167 oncologists. Sixty-four per cent of them practiced in North America and Europe, and the remaining practiced in non-Western countries. In disclosing the cancer diagnosis and prognosis, physicians from Western countries were less likely to withhold unfavorable information from the patient at the family's request, avoid the discussion entirely, or use euphemisms. Another questionnaire given to both physicians and patients in the US and Japan confirmed this finding and revealed that both physicians and patients in the West are more inclined toward respecting patient autonomy and informed consent.^7^ In that questionnaire sample, groups of Japanese physicians (n=400) and patients (n=65) as well as US physicians (n=120) and patients (n=60) were selected randomly. A majority of both US physicians and patients, but only a minority of Japanese physicians and patients, agreed that a patient should be informed of an incurable cancer diagnosis before their family is informed.

There is now a clear focus, both legally and ethically, on the issue of informed consent and patient autonomy. It is now an unshakable belief that telling the truth is a moral duty and that the patient has a need to know the truth to make decisions.

The change in the attitude in the west over the 1950s and 1960s was multifactorial in etiology. Revelation of the post World War II Nuremberg trials that disclosed experimentation on humans without consent, showed the need for the legal and ethical importance of informed consent.

The 1950s and 1960s was the era of social upheaval in the US, when movements for human rights were demanding rights for women, consumers, and finally patients, who began to demand to be fully informed about their diagnosis, prognosis, and treatment options. Furthermore, the increased optimism about the cure of some cancers due to advances in both surgical and radiation oncology and the beginning of medical oncology gave more treatment options and increased survival in many cancers. This in turn provided the momentum toward disclosing diagnoses to patients. On the physicians' side, there was greater recognition of communication as an effective means of enhancing patient understanding and compliance.

## Saudi perspective

Attitudes are undergoing steady, albeit slow changes in many non-Western nations, including Saudi Arabia. Public education, in addition to the partial cultural openness due to the communication revolution, and worldwide globalization are having some effect in changing a few aspects of the sociocultural atmosphere in Saudi Arabia. Because of the complicated political, social and religious mix of values that govern the Saudi society, changes, if allowed to happen, are slow and follow a cautious path.

While the values of patient autonomy and informed consent are now rooted deep in the conscience of Western societies and control and shape the physician-patient relationship, these values are not prominent yet and have not become so influential in the Saudi society. The patient can frequently be thought of as an extended individual, with family members intimately involved with shared decision-making responsibility. Family members, and to a lesser extent friends, find themselves forced by their genuine cultural and mainly religious values to extend their help and support to their relatives or friends. They find themselves obliged, and frequently, allowed by the patient to do so by stepping forward and taking over some or all of the patient's responsibilities. This is the way they show sympathy and support to the sick relative or friend. They basically believe that patients (especially female patients) are very vulnerable and should not be left alone to handle the stress of knowing the bad news or the stress of making decisions. This supportive attitude, unfortunately, at the end may evolve into a dominating attitude that steals the patient's basic right of knowledge and decision-making. Nevertheless, many patients (especially old women) accept this situation, where the dominating relatives (sons, most of the time), become the major players. These patients trust their dominating relatives and literally hand over some or all responsibilities.

Bedikian *et al*. conducted a survey of 100 adult patients and companions referred to the Department of Oncology at King Faisal Specialist Hospital, Riyadh in 1984.^8^ He found that only 16% of patients were told that they had “cancer” and 34% were told they had a “tumor”. On the other hand, 69% of companions were told about the diagnosis of cancer. Another questionnaire was conducted almost 10 years later to assess the physician attitudes towards sharing information and decision-making with patients in the setting a serious illness.^9^ Two hundred and forty-nine physicians from three different areas of the country participated in the study. Seventy-five per cent of physicians preferred to talk with close family members rather than patients.

In the face of such an attitude, oncologists from King Faisal Specialist Hospital, Riyadh,^10^ tried to explain why physicians avoid telling their patients about their disease. They concluded that physicians may not know what to say or how to say it when they are about to break bad news. They may wish to avoid the difficulties of having to cope with a patient who is disturbed by the bad news, they may feel the patient simply will not cope, or it may take too much time and patience out of a busy work schedule.

To further monitor over the years for any evolving change in attitudes, a recent study was conducted to assess physician and public views in the country towards involving the patient versus the family in the process of diagnosis disclosure and decision-making. The study surveyed 321 physicians and 264 hospital attendees from six different regions.^11^ In the case of a patient with incurable cancer, 67% of doctors and 51% of hospital attendees indicated that they would inform the patient in preference to the family of the diagnosis (*P*=.001). Assuming the family already knew, 56% of doctors and 49% of hospital attendees would tell the patient even if the family objected (difference not statistically significant). However, in the case of HIV infection, 59% of physicians and 81% of hospital attendees would inform the family about HIV status without the patient's consent (*P*=.001). The authors concluded that there is a need for greater recognition of patient autonomy among physicians.

These studies only assessed public and physician preferences and attitudes, which are clearly conservative regarding disclosure. There was at least one study that was conducted to assess patient preference specifically towards disclosure of diagnosis and prognosis.^12^ A small survey of 114 patients in a teaching hospital in the Eastern Province revealed that almost all (113 patients) wished to know all information about their cancer and only one patient preferred to know partial information. All patients were against withholding information. Almost all patients wanted to know the benefits and adverse effects of therapy (98% and 99%, respectively), and all wanted to know about the prognosis of their disease. This study showed how the patient's preference is toward absolute disclosure while the public attitude is, in general, against full disclosure.

## Current dilemma: Easy to say, difficult to do!

Every healthcare provider involved in the care of cancer patients knows how difficult it can be to disclose the diagnosis to the patient. Disclosure of prognosis, especially after failure of therapy, is even more difficult. To overcome such a difficulty, the following suggestions are provided:

- Disclosure has to be a systematic process and has to follow guidelines for breaking bad news. These guidelines are set up to make the process of disclosure smooth and fruitful. They should be taught in medical school and be a part of postgraduate training.
- Establishment of a support program for both patients and their families. There is no doubt that breaking bad news is a daily routine in oncology practice, putting a continuous psychological and emotional burden on physicians. Establishing a support program will definitely help physicians to cope with the situation better and enable them to perform the task in the optimal way.
- The argument for disclosure can be supported by many strong points, including the clear Islamic perspective to respect the patient and protect the patient's right to know and to freely make choices. It can also be supported by studies about patient preferences, which include evidence of benefit that is now undebatable.^12^ These benefits include building a trustful relationship with the physician, ability to make decisions or at least share in decision-making, improving compliance, and last but not the least, planning the end of life.
- More local research is needed to study all public, patient, and physician attitudes as well as signs of change and the underlying factors influencing change.

Finally, one of the most appropriate approaches that fit patients in many developing countries, including Saudi Arabia, is what James Hallenbeck and Robert Arnold described in an article titled “A Request for Nondisclosure: Don't Tell Mother”.^13^ This model of negotiation was used with the family and found to be very useful in the Saudi situation. This is a summary of the key points with minor modifications to fit the local clinic practice:

- Unlike in many parts in the world, physicians in Saudi Arabia, as per the local sociocultural background, frequently need to establish a physician-family rapport in addition to a physician-patient rapport.
- Respect the sociocultural background. Most of the time the family would like to know first. This can be acceptable provided that the patient is not denied the right to know.
- Try to understand the family's viewpoint and respond empathetically to their distress, keeping in mind that the aim is not to hide any information from the patient upon his or her request. Explain to them the benefits to the patient from disclosure and the practical difficulties associated with not telling the diagnosis.
- Explain the importance of truthfulness for you and negotiate how you will respond if the patient asks to be told the truth. Stress the point that, “if he or she asks me to tell the truth, I must do so”.
- Talk to the family about what the patient would want and explain that “you are fine with a family member to be the decision-maker”, if the patient concurs with the decision.
